# The Impact of Phytases on the Release of Bioactive Inositols, the Profile of Inositol Phosphates, and the Release of Selected Minerals in the Technology of Buckwheat Beer Production

**DOI:** 10.3390/biom10020166

**Published:** 2020-01-21

**Authors:** Robert Duliński, Marek Zdaniewicz, Aneta Pater, Dagmara Poniewska, Krzysztof Żyła

**Affiliations:** 1 Department of Biotechnology and General Technology of Food, Faculty of Food Technology, Agricultural University in Krakow, Balicka 122, 30-149 Krakow, Poland; dagmara.poniewska@urk.edu.pl (D.P.);; 2 Department of Fermentation Technology and Microbiology, Faculty of Food Technology, Agricultural University in Krakow, Balicka 122, 30-149 Krakow, Poland; m.zdaniewicz@urk.edu.pl (M.Z.); a.pater@urk.edu.pl (A.P.)

**Keywords:** D-*chiro*-inositol, *myo*-inositol, buckwheat beer, HPAEC-PAD, inositol phosphates profile

## Abstract

A relatively high concentration of phytate in buckwheat malt, and the low activity of endogenous buckwheat phytases, both of which limit the effective use of substrates (starch, proteins, minerals) for fermentation and yeast metabolism, gives rise to the potential for application of phytases in beer production. This study aims at obtaining a 100% buckwheat wort with high bioactive cyclitols (*myo*-inositol and D-*chiro*-inositol) concentrations released by exogenous phytases and acid phosphatases. Two mashing programs were used in the study, i.e., (1) typical for basic raw materials, namely the well-established Congress method, and (2) optimized for phytase activity. The results indicated a nearly 50% increase in the level of bioactive *myo*-inositol and an 80% degradation of phytate in the wort as a result of simultaneous application of phytase and phosphatase enzymes in the mashing of buckwheat malt. In addition, high D-*chiro*-inositol concentrations were released from malt to the buckwheat wort. The concerted action of the two phytases significantly increased (19–44%) Zn^2+^ concentrations in wort. This may be of great importance during mash fermentation by *Saccharomyces cerevisiae* yeasts. There is a potential to develop technology for buckwheat beer production, which, in addition to being free from gluten, comprises high levels of bioactive *myo-* and *D-chiro*-inositols.

## 1. Introduction

The pseudocereal Buckwheat (*Fagopyrum esculentum*) is attracting increasing interest as a raw material for functional food production. Due to its unique nutritional value comprising high concentrations of phytosterols, flavonols, vitamin binding proteins, a desirable fatty acid profile, and an advantageous amino acid composition, buckwheat may be used in gluten-free diets, as an ingredient in bread production [1,2,3,4,5,6], and for manufacturing of gluten-free beer. The first tests carried out on buckwheat beer, including biochemical characterization and sensory analyses, confirmed that after process optimization it could have potential for marketing [7]. The high phytate level in the initial raw material, however, is among the detrimental factors that limit the application of buckwheat in fermented food and beverages [8]. There have been attempts to introduce genetically-modified varieties of buckwheat that are low in phytate content into brewing technology [9].

Phytic acid (*myo*-inositol 1,2,3,4,5,6-hexakisphosphate, InsP_6_) is the main phosphorus reservoir in plant tissues, which, in the form of phytate salts, complexes with proteins and carbohydrates, and is considered as an antinutritional factor [10,11]. Phytate, as the compound that reduces the bioavailability of minerals, protein, and starch, may exert a negative effect on mashing and fermentation processes. It decreases starch degradation and the available spectrum of fermentable sugars mainly by chelating Ca^2+^ and lowering α-amylases activity, inhibits protease, and reduces free amino acid release, as well as chelates cofactors like Mg^2+,^ Zn^2+^ ions, which are crucial for proper *Saccharomyces cerevisiae* fermentation [9,10,12]. Only limited phytate dephosphorylation may occur by means of thermal hydrolysis, e.g., during cooking, and therefore, enzymatic decomposition seems to be the only effective treatment. The following enzymes that differ in substrate specificity during the hydrolysis of *myo*-inositol phosphate esters have been distinguished: 3-phytase A (EC.3.1.3.8), 6-phytase A (EC.3.1.3.26), and phytase B (EC.3.1.3.2) [13]. Firstly, 3-phytase A starts the catalytic action from the phosphate group at carbon C3 of the *myo*-inositol ring, while 6-phytase A starts phosphate hydrolysis at carbon C6. Phytases A are not able to hydrolyze the bond at carbon C2 due to its axial conformation. This bond, however, can be hydrolyzed by phytase B, i.e., the nonspecific acid phosphomonoesterase [13]. Phytases are known to be synthetized in plants tissues (i.e., endogenic enzymes that are active during soaking and sprouting of seeds), bacteria, and filamentous fungi [14,15]. Phytate dephosphorylation that results from plant endogenous phytase action in barley, wheat, rye, and particularly, in buckwheat grain tissues during mashing is limited, mainly due to process conditions which are out of the optima for pH (4.5–5.5) and temperature values (45–65 °C) [13]. Commercial preparations of phytases have been successfully used in the animal feed industry for a few decades [16]. However, despite their vast potential in the food production and food processing industry, no product enriched with these enzymes has found its way to the market. In this study, commercial 6-phytase A, 3-phytase A, and an acid phosphatase (phytase B) preparations were used. Although the application of phytases in the mashing of alternative raw materials, such as maize and sorghum, has already been studied [17], no attempts to apply a combination of phytase A and phytase B in the technology of buckwheat beer manufacturing are known to the authors. In a previous study, we reported the complete release of D-*chiro*-inositol (DCI) from buckwheat malt, but only 11–13% of the total *myo*-inositol (MI) content was released to the wort [18]. The purpose of the current study was to exploit the possibilities of the enzymatically-aided release of buckwheat *myo*-inositol to prepare a wort that, in addition to being gluten-free, is also high in both bioactive isomers of inositols [19,20]. Dinicola with coworkers [21] provided a mechanism of MI depletion by high glucose concentrations in nervous tissues by the activation of the glucose-sorbitol pathway, whereby glucose is first converted to sorbitol by aldose reductase, and then to fructose by sorbitol dehydrogenase. This raises the intracellular osmolarity and inhibits the uptake of other osmolytes like inositols. MI depletion may worsen insulin resistance and diabetes complication, including disturbances in cellular redox, and free radical defense, increased oxidative glycation stress, and renal function. Conversely, MI supplementation improves several diabetes symptoms. Moreover, DCI deficiency was also found to be closely related to insulin resistance [22]. There is growing experimental evidence to show the rationale behind applying both isomers of inositol to prevent and cure diabetes and diabetic complications [23,24].

## 2. Materials and Methods

### 2.1. Materials

Château Buckwheat malt (crop year 2018) was produced by Castle Malting^®^ company (Lambermont, Belgium). According to the manufacturer’s specifications, the moisture content was up to 5%, the extract min. 66%, the protein content up to 11%, and color in the range of 4–15 EBC. The producer of Château Buckwheat malt recommends this malt (up to 40% of the mix) for the production gluten-free beers or/and specialty beers. Trace amounts of other cereals, e.g., containing gluten, may be found in the malt. Buckwheat malt adds a nutty and malty flavor to beer. However, this malt has no diastatic power. Before the preparation of laboratory wort, buckwheat malt was stored in a cool and clean environment.

### 2.2. Enzymes

#### 2.2.1. Amylase

In the study, the MATS^®^ L CLASSIC enzyme, which is a liquid thermostable alpha-amylase obtained from a nongenetically modified strain of *Bacillus licheniformis*, was purchased from DSM Nurtitionals, (TE Heerlen, the Netherlands) and applied at 0.4 g/kg. This enzyme was added at the beginning of mashing following the recommendations of the supplier.

#### 2.2.2. Phytases and Acid Phosphatase

The preparation of 3-phytase A (Natuphos^®^) was obtained from BASF, Ludwigshafen, Germany, while 6-phytase A (Ronozyme^®^ NP) was a product of Novozymes, Bagsværd, Denmark, and phytase B (Finase^®^ AP) was from AB Enzymes, *Rajamäki*, Finland. Acid phosphatase activity was determined at 40 °C by incubating the enzyme with 5.5 mM solution of sodium p-nitrophenyl phosphate at a pH of 4.5 for 15 min. One unit of acid phosphatase activity (AcPU) was defined as the amount of enzyme which, under the reaction conditions, releases 1 μmol of *p*-nitrophenol in 1 min [25]. The measured activity of acid phosphatase in Finase^®^ AP was 13.2 kAcPU/g. One unit of phytase A activity (FTU) was defined as the amount of enzyme that releases 1 μM of inorganic phosphorous from 2 mM of sodium phytate in 1 min at 40 °C, at a pH of 4.5 [25]. The measured activity of 3-phytase A was 5.1 kFTU/g, and that of 6-phytase A was 4.7 kFTU/g. Finase P was added to the buckwheat malt at a ratio of 15 mg/g, Ronozyme at a ratio of 30 mg/g, and Finase AP at a ratio of 5 mg/g.

### 2.3. Preparation of Laboratory Worts

#### Mashing

Buckwheat malt brews were prepared in Mash Bath R12 with connection to PC (1-CUBE s.r.o., Havlíčkův Brod, Czech Republic) similarly to the method of handling barley malt (EBC 4.5.1). For this purpose, 50 g of buckwheat malt milled in a laboratory grinder was weighed into tarred mash containers, which were then placed in a water-heated apparatus at 45 °C. Agitators were set up and the “Congress program” was selected. Next, 200 mL of distilled water at 45 °C was poured in portions into the containers. The apparatus was held at 45 °C for 30 min. Then, the temperature was raised at a rate of 1 °C/min until it reached 70 °C, with constant stirring of samples. In order to ensure the highest efficacy of the enzyme, the temperature was set at this level according to the work of Qian and Kuhn [26]. When the apparatus reached 70 °C, 100 mL distilled water was warmed to the same temperature and added to the cups, and then the set temperature was maintained for 1 h. Next, the containers were cooled to 20 °C and filled with distilled water up to a mass of 450.0 g.

Optimal is a mashing program developed in order to optimize temperature ranges for the activity of phytases. The apparatus maintained a temperature of 35 °C for 15 min, 45 °C for 15 min, 65 °C for 40 min, 72 °C for 30 min, and 78 °C for 10 min. Next, the temperature was lowered at a rate of 1 °C/min until it reached 70 °C, with constant stirring of samples. When the apparatus reached 70 °C, 100 mL of distilled water warmed to the same temperature was added to the cups, and the set temperature was maintained for 1 h. Then, the containers were cooled to 20 °C and filled with distilled water up to the mass of 450.0 g.

### 2.4. Inositol Analysis

The analysis of *myo*- and D-*chiro*-inositol was carried out based on the method described by Duliński et al. [27]. The determination of the total inositol content in the raw material was conducted following the procedure set out in Duliński et al. [28]. Standards of *myo*-inositol (99% purity) and D-*chiro*-inositol (98% purity) for HPLC methods were purchased from Sigma-Aldrich. The correlation coefficient for the example standard curve in inositols analysis amounted to 0.998 (for *myo*-inositol).

### 2.5. Phytate Analysis

A Thermo-Dionex Ultimate 3000 system attached to an ED50a electrochemical detector equipped with a conductivity cell (Thermo-Dionex, Sunnyvale, CA, USA) was used for the High-Performance Anion-Exchange Chromatography (HPAEC) analysis. Briefly, samples of wort diluted at a 1:10 ratio with deionized water were separated using an Omnipac Pax-100 anion-exchange column (250 × 4 mm I.D.) connected to an Omnipac Pax-100 (8 × l mm) guard column in a series (Thermo-Dionex, Sunnyvale, CA, USA). A gradient mobile phase using 200 mM sodium hydroxide (A), deionized water (B), and water–isopropanol (50:50, *v/v*) (C) was applied. An anion micromembrane suppressor AMMS 300 4-mm (all from Thermo-Dionex, Sunnyvale, CA, USA) system was used to suppress the mobile phase conductivities before entering the conductivity cell (regenerant 0.25 M sulfuric acid) according to Thermo-Dionex Application Note 65 (2003) [29]. The correlation coefficient for the example standard curve for phytate analysis amounted to 0.957.

### 2.6. Semi-Qualitative Analysis of the Inositol Phosphate Profile

#### 2.6.1. Purification of the Inositol Phosphates Using Ion-Exchange Chromatography

A 15 mL sample of wort was diluted at 1:1 with distilled water. Low-pressure ion-exchange liquid chromatography was performed to separate *myo*-inositol phosphates from the extract. Chromatography columns were filled with 2 g of AG 1 × 8 anion-exchanger (200–400 mesh, chloride form) (Bio-Rad, Hercules, CA, USA) and conditioned with 10 mL of deionized water. Next, 30 mL of the diluted buckwheat wort was passed through the column, and the column was rinsed with 10 mL of deionized water. Elution of *myo*-inositol phosphates was performed by 20 mL of 2 M hydrochloric acid. Eluates were evaporated on a water bath preheated to 40 °C and redissolved in 5 mL of deionized water, frozen at -18 °C, and stored for HPLC analysis.

#### 2.6.2. HPLC Analysis

The profile of the isomers of *myo*-inositol phosphates was analyzed by an analytical system using high-pressure anion-exchange chromatography (HPAEC) with postcolumn derivatization and spectrophotometric detection [30]. Before injection into a chromatographic column, samples were filtered through a syringe filter (0.45 μm). A reference sample for the identification of peaks was prepared by dissolving 2.3 g of sodium phytate in 50 mL of deionized water and adjusting the pH to 4.0 by 2 M HCl. The solution was autoclaved for 40 min at 121 °C under 1 atm. The elution sequence of different isomers was established according to the work of Blaabjerg et al. [30] using the appropriate standard solutions, i.e., sodium phytate (InsP_6_, 96% purity), Ins(1,2,4,5,6)P_5_ (96% purity), Ins(1,4,5,6)P_4_ (95% purity), Ins(1,3,4,5)P_4_ (>90% purity), Ins(1,4,5)P_3_ (95% purity), Ins(1,3,4)P_3_ (90% purity), and *myo*-inositol 2-monophosphate (~95% purity) (all purchased from Sigma-Aldrich, Steinheim, Germany).

### 2.7. Determination of Metal Ions

The contents of metals in the samples of buckwheat wort were determined by atomic absorption spectrometry with the flame atomization technique (Varian AA240FS), using an automatic dispensing sample system (SIPS–20) (Agilent, Santa Clara, CA, USA). The flows of gas (acetylene) and air were 3.5 and 14 L/min, respectively. Before analysis, the samples were subjected to a process of wet mineralization with the addition of 4 mL of concentrated HNO_3,_ in sealed pressure vessels using a microwave oven Mars Xpress (1200 W, 170 °C, 15 min; CEM Corp., Matthews, NC, USA). The elements were determined using a single sample aspiration via a rapid sequence mode (called Fast Sequential). Standard solutions of cations were prepared according to MERCK standards (1000 mg·L^−1^) (Merck, Bilerica, MA, USA).

### 2.8. Statistical Analysis

Experimental data were subjected to the one-way analysis of variance (ANOVA) to detect significant differences among means and expressed as a mean ± standard deviation (SD). Differences among means were checked by the Tukey test at *p* < 0.05 using the Statistica for Windows, version 12.5 (Statsoft Inc., Tulsa, OK, USA) statistical software.

## 3. Results and Discussion

### 3.1. Release of Bioactive Inositols

The first series of experiments involved the introduction of 3- and 6-phytase A (Ronozyme and Finase P, respectively) into malt during mashing. In comparison to the control wort (62.08 μg/mL), no statistically significant increase in MI was observed, either in the case of Congress mashing (68.01–71.11 μg/mL) or in the program optimized for phytase activity (66.7 μg/mL) (Table 1). A similar observation was made with D-*chiro*-inositol, whose content did not change significantly in the samples of mashed malt with added phytases, either in the mashing variant optimized for phytase activity (211.07 and 188.83 μg/mL for 3- and 6-phytase, respectively) or in the Congress mashing (201.38 and 223.19 μg/mL). In this case, due to the stereospecificity of the applied enzymes that eventually release inositol from phytate complexes in the *myo-* configuration, no significant changes could have been expected. The reported D-*chiro*-inositol concentrations are comparable with the DCI contents (278.3–381.7 μg/mL) that were observed previously in buckwheat wort treated only with standard amylolytic enzymes (Brewers Compass, and Mats L Classic) [18].

The simultaneous application phytase and acid phosphatase at the mashing stage contributed to the increase in concentration of *myo*-inositol by nearly 50% from 62.08 μg/mL to 92.66 μg/mL for mashing under conditions optimized for phytase action (Table 1). This suggests that the cooperation of both phytase A and B, but not phytase A alone, may be efficacious in effecting MI release from buckwheat malt during mashing. In different applications, but mainly in poultry nutrition, the simultaneous supplementation of phytase A and B was found to be superior to single phytase A [31]. On the other hand, it should be noted that despite high dosages of phosphorolytic enzymes (Finase AP at 10 U/g of malt), the pool of the released bioactive MI increased only to around 20% of its concentration in the buckwheat malt. The remaining 80% was most probably MI deposited in the form of phospholipids or other complexes unavailable for the catalytic action of the phosphorolytic enzymes [11]. Further research seems necessary to optimize MI release from buckwheat malt, since buckwheat wort contains high amounts of both bioactive inositol isomers.

### 3.2. Phytate and Inositol Phosphates Profile

The most effective phytate degradation process was observed when a combination of 3-phytase A and acid phosphatase was applied. This variant allowed for a nearly 80% reduction in phytate content, i.e., from 2.74 mg/g for the control to 0.55 mg/g of buckwheat malt, after mashing with the temperature optimized for phytases. The 3-phytase A preparation used individually in both mashing programs was more effective than 6-phytase A (0.72 mg/g—Finase P and 1.24 mg/g of Ronozyme), although in the Congress mashing program, this difference was not statistically significant (1.09 and 1.14 mg/g of malt) (Table 2). There were also no statistically significant differences between the content of phytate in the wort supplemented with 3-phytase (Finase P) and the wort with both 3-phytase and acid phosphatase (Finase AP) (1.09 mg/g and 0.99 mg/g of malt, respectively). Mikulski with coworkers [17] reported that the introduction of phytase at 8 U/g of maize malt under conditions of high gravity mashing resulted in complete phytate dephosphorylation [14], but these researchers used different mashing conditions (temperature 55 °C, pH 5.5) that were more adjusted for phytase activity. In our study, the pH was kept at the standard value for mashing (5.8) to ensure the effective action of bacterial α-amylase that is crucial for the proper mashing of buckwheat malt. In another study, beneficial phytase effects applied at 0.8–1.2 U/g resulted from the reduced mashing time [32].

**Figure 1:**
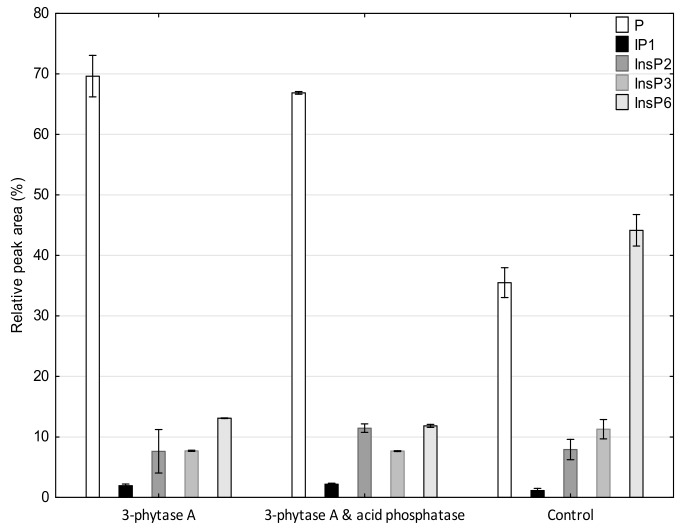
Profile of the inositol phosphates by HPAEC–UV/Vis chromatography on CarboPackPA1 column obtained from the analysis of the buckwheat wort using the mashing program optimized for the activity of phytases.

**Figure 2:**
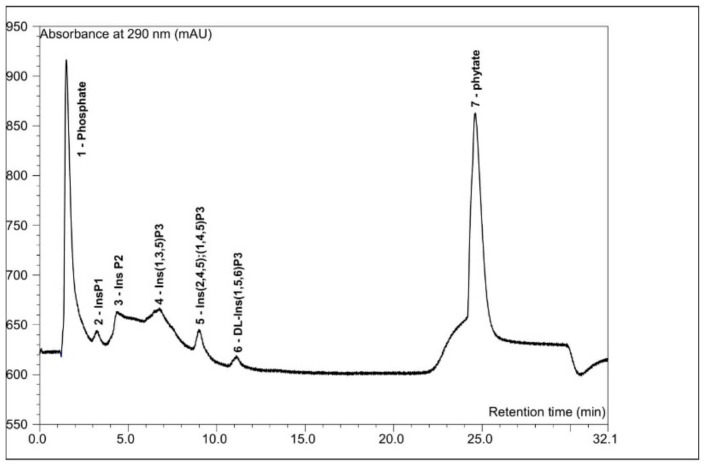
Sample chromatogram from the profile in the control buckwheat wort analyzed by HPAEC–UV/Vis chromatography.

The analysis of inositol phosphate profiles conducted using anion-exchange, high-performance liquid chromatography with postcolumn modification and UV/V is detection allowed us to identify not only phytate, but also lower phosphates of *myo*-inositol. In the control sample, where mashing was conducted without phosphorolytic enzymes, the dominant peak was represented by InsP_6_ (45% of the total area, 5 mg/g of malt) (Figure 1), with a significant share of InsP_1–2_ fractions (35%). Compared to raw buckwheat seeds, the process of steeping, malting, and mashing affected the redistribution of the profile towards higher concentrations of lower inositol phosphates, and resulted in a 75% reduction in the InsP_6_. In the group of intermediate inositol phosphates, Ins(1,2,5,6)P_4_ and Ins(1,5,6)P_3_ were identified in the wort mashed using the Congress method, which agrees well with the specificity of 3-phytase A (Figure 2).

It is worth noting that the addition of acid phosphatase did not significantly influence the decrease in phytic acid content (Table 2), most probably due to higher affinity of the enzyme to lower *myo*-inositol esters. It should be noted, however, that in the mash optimized for phytase activity, a 10% increase in InsP_1–2_ and an increase in the concentration of free *myo*-inositol were observed in wort (Table 1). In the profile of inositol phosphates observed in both the control and the phytase supplemented wort, except for the main peak of phytic acid, which constituted from 13% (exogenous phytases) to 43% (control without phosphorolytic enzymes) of the total peak area, no significant concentrations of InsP_4–5_ were observed (Figure 1 and Figure 2). The applied HPAEC-UV/Vis technique also allowed us to semiquantitatively monitor the level of inorganic phosphorus (P), which, apart from MI, is another final product of phytate hydrolysis performed in wort by phosphorolytic enzymes. The increase in the P concentrations ranged from 38% of the relative peak area of this compound (control) to 67% (3-phytase A) in the optimal variant of mashing (Figure 2), confirming an effective enzymatic degradation of the phytate to one of the end products.

### 3.3. Mineral Availability

Compared to classical barley malt (0.3 μg/mL), the content of Zn^2+^ ions determined in buckwheat wort was approximately 70% lower (1.012–1.58 μg/mL) [33]. The analysis of zinc ion content in buckwheat wort indicated a significant effect of phytase addition on the increase of their concentrations in the liquid after mashing. This phenomenon was observed in the program optimized for phytase activity, where Zn^2+^ contents increased from 19% (3-phytase A) to 44% (6-phytase A), as compared to the control (1.02 μg/mL) (Table 2). Similar observations were also made in the standard mashing program. In the Congress version of mashing, the content of zinc ions increased from 1.12 μg/mL (control mush) to 1.25 μg/mL (3-phytase A) and 1.58 μg/mL (6-phytase A). The application of acid phosphatase along with 3-phytase A did not exert a significant effect on the amounts of zinc in the wort (1.43 μg/mL and 1.26 μg/mL for the optimal and congress versions, respectively) (Table 2).

Phytate is one of the most potent chelators of zinc ions [34]. Therefore, an enzymatic dephosphorylation of this compound of up to 55–80% had a significant influence on the concentrations of these ions in the buckwheat wort. This may be of importance during wort fermentation with *Saccharomyces cerevisiae* cultures, for which Zn^2+^ serves as a cofactor of many enzymes involved in glycolysis, alcohol dehydrogenases [35,36], and in the cell response to stress induced by increased ethanol concentrations [33]. Muy-Rangel et al. reported that about 80% of zinc ions were consumed by yeasts during fermentation, and recorded relatively low zinc concentrations in the final beer [37].

In the case of other analyzed divalent ions (Ca^2+^, Mg^2+^) that are known to form complexes with InsP_6_ [38], no significant influence of phytase supplementation on their contents in wort was observed (Table 2).

## 4. Conclusions

Study of the production of functional buckwheat beer with increased concentrations of bioactive cyclitols, namely *myo*-inositol and D-*chir*o-inositol, revealed that up to 80% of buckwheat phytates may be hydrolyzed during mashing. The simultaneous application of 3-phytase A and acid phosphatase (phytase B) yielded a 50% increase in the *myo*-inositol concentration in the wort (93 μg/mL), but this accounted for only 20% of the total *myo*-inositol concentration in malt. In addition, high D-*chiro*-inositol levels (229 μg/mL) in buckwheat wort were accompanied by a 44% increase in Zn^2+^ ion concentrations (1.58 μg/mL), which is expected to stimulate *S. cereviasiae* metabolism and cell activity during wort fermentation.

## Figures and Tables

**Table 1:** Bioactive inositols contents (µg/mL) in buckwheat wort.

Enzyme(s)	Mashing					
	Optimal			Congress		
	MI	% of Total MI	DCI	MI	% of Total MI	DCI
3-phytase A	66.74 ± 2.33 ^a^	13.8	211.07 ± 12.14 ^a^	71.11 ± 6.04 ^b^	14.9	201.38 ± 9.41 ^a^
6-phytase A	66.78 ± 5.48 ^a^	13.9	188.83 ± 13.71 ^a^	68.01 ± 1.91 ^ab^	14.2	223.19 ± 31.9 ^a^
3-phytase and acid phosphatase	92.66 ± 6.37 ^b^	19.4	229.76 ± 10.61 ^a^	72.85 ± 7.71 ^b^	15.2	211.84 ± 8.09 ^a^
Control	62.08 ± 1.92 ^a^	13.0	210.99 ± 27.85 ^a^	61.66 ± 2.68 ^a^	12.9	205.35 ± 16.21 ^a^

Different superscript letters in the same column indicate significant differences (*p* < 0.05). Abbreviations: MI—*myo*-inositol and DCI—D-*chiro*-inositol.

**Table 2:** Phytate (mg/g malt) contents and selected mineral elements availability (μg/mL) in buckwheat wort supplemented with phytases.

Mashing	Enzyme(s)	InsP_6_	% of Reduction	Mg	Zn	Ca
Optimal	3-phytase A	0.72 ± 0.29 ^b^	74	124.68 ± 3.94 ^a^	1.19 ± 0.21 ^b^	14.08 ± 2.07 ^a^
	6-phytase A	1.24 ± 0.08 ^c^	55	129.37 ± 5.19 ^a^	1.44 ± 0.11 ^b^	15.16 ± 1.02 ^a^
	3-phytase and acid phosphatase	0.55 ± 0.12 ^a^	80	135.26 ± 6.17 ^a^	1.43 ± 0.15 ^b^	14.08 ± 0.89 ^a^
	Control	2.741 ± 0.12 ^d^	-	125.71 ± 10.16 ^a^	1.02 ± 0.14 ^a^	16.12 ± 1.41 ^a^
Congress	3-phytase A	1.09 ± 0.14 ^a^	64	134.15 ± 13.91 ^a^	1.25 ± 0.11 ^b^	14.38 ± 2.21 ^a^
	6-phytase A	1.14 ± 0.12 ^a^	63	148.83 ± 8.20 ^a^	1.58 ± 0.13 ^c^	15.69 ± 2.50 ^a^
	3-phytase and acid phosphatase	0.99 ± 0.07 ^a^	68	144.06 ± 6.78 ^a^	1.26 ± 0.15 ^b^	11.45 ± 2.1 ^a^
	Control	3.075 ± 055 ^b^	-	145.21 ± 4.99 ^a^	1.12 ± 0.01 ^a^	18.81 ± 4.29 ^a^

Different superscript letters in the same column indicate significant differences (*p* < 0.05).

## References

[1] Starzynska-Janiszewska A., Dulinski R., Stodolak B., Mickowska B., Wikiera A. (2016). Prolonged Tempe-Type Fermentation in Order to Improve Bioactive Potential and Nutritional Parameters of Quinoa Seeds. J. Cereal Sci..

[2] Rubio-Flores M., Serna-Saldivar S.O. (2016). Technological and Engineering Trends for Production of Gluten-Free Beers. Food Eng. Rev..

[3] Tang C., Gong Q., Sun X. (2008). Functional Properties of Buckwheat (*Fagopyrum esculentum* Moench) Seed Protein Isolate: Effects of Limited Enzymatic Hydrolysis with Trypsin. Eur. J. Plant Sci. Biotechnol..

[4] Gimenez-Bastida J.A., Piskula M., Zielinski H. (2015). Recent Advances in Development of Gluten-Free Buckwheat Products. Trends Food Sci. Technol..

[5] Giménez-Bastida J.A., Piskula M.K., Zieliñski H. (2015). Recent Advances in Processing and Development of Buckwheat Derived Bakery and Non-Bakery Products—A Review. Polish J. Food Nutr. Sci..

[6] Nepali B., Bhandari D., Shrestha J. (2019). Mineral Nutrient Content of Buckwheat (Fagopyrum Esculentum Moench) for Nutritional Security in Nepal. Malaysian J. Sustain. Agric..

[7] Phiarais B.P.N., Mauch A., Schehl B.D., Zarnkow M., Gastl M., Herrmann M., Zannini E., Arendt E.K. (2010). Processing of a Top Fermented Beer Brewed from 100% Buckwheat Malt with Sensory and Analytical Characterisation. J. Inst. Brew..

[8] Duliński R., Starzyńska-Janiszewska A., Byczyński Ł., Błaszczyk U. (2017). Myo-Inositol Phosphates Profile of Buckwheat and Quinoa Seeds: Effects of Hydrothermal Processing and Solid-State Fermentation with Rhizopus Oligosporus. Int. J. Food Prop..

[9] Edney M.J., Rossnagel B.G., Raboy V. (2007). Effect of Low-Phytate Barley on Malt Quality, Including Mineral Loss, during Fermentation. J. Am. Soc. Brew. Chem..

[10] Balk J., Connorton J.M., Wan Y., Lovegrove A., Moore K.L., Uauy C., Sharp P.A. (2019). Improving Wheat as a Source of Iron and Zinc for Global Nutrition. Nutrition.

[11] Bohn L., Meyer A.S., Rasmussen S.K. (2008). Phytate: Impact on Environment and Human Nutrition. A Challenge for Molecular Breeding. J. Zhejiang Univ. Sci. B.

[12] Frontela C., Ros G., Martínez C. (2011). Phytic Acid Content and “In Vitro ” Iron, Calcium and Zinc Bioavailability in Bakery Products: The Effect of Processing. J. Cereal Sci..

[13] Konietzny U., Greiner R. (2002). Molecular and Catalytic Properties of Phytate-Degrading Enzymes (Phytases). Int. J. Food Sci. Technol..

[14] Avendano K.A., Anguiano M., Lopez C.E., Montanez L.E., Sifuentes L., Balagurusamy N. (2016). Microbial Enzymes Applications in Food Processing. Agro Food Ind. Hi. Tech..

[15] Azeke M.A., Greiner R., Jany K.D. (2011). Purification and Characterization of Two Intracellular Phytases from the Tempeh Fungus Rhizopus Oligosporus. J. Food Biochem..

[16] Haefner S., Knietsch A., Scholten E., Braun J., Lohscheidt M., Zelder O. (2005). Biotechnological Production and Applications of Phytases. Appl. Microbiol. Biotechnol..

[17] Mikulski D., Kłosowski G., Rolbiecka A. (2015). Influence of Phytase and Supportive Enzymes Applied during High Gravity Mash Preparation Onthe Improvement of Technological Indicators Ofthe Alcoholic Fermentation Process. Biomass Bioenergy.

[18] Duliński R., Zdaniewicz M., Pater A., Żyła K. (2019). Impact of Two Commercial Enzymes on the Release of Inositols, Fermentable Sugars, and Peptides in the Technology of Buckwheat Beer. J. Am. Soc. Brew. Chem..

[19] Valluru R., Van den Ende W. (2011). Myo-Inositol and beyond--Emerging Networks under Stress. Plant Sci..

[20] Croze M.L., Soulage C.O. (2013). Potential Role and Therapeutic Interests of Myo-Inositol in Metabolic Diseases. Biochimie.

[21] Dinicola S., Minini M., Unfer V., Verna R., Cucina A., Bizzarri M. (2017). Nutritional and Acquired Deficiencies in Inositol Bioavailability. Correlations with Metabolic Disorders. Int. J. Mol. Sci..

[22] Garzon S., Laganà A.S., Monastra G. (2019). Risk of Reduced Intestinal Absorption of Myo-Inositol Caused by D-Chiro-Inositol or by Glucose Transporter Inhibitors. Expert Opin. Drug Metab. Toxicol..

[23] Tahir F., Majid Z. (2019). Inositol Supplementation in the Prevention of Gestational Diabetes Mellitus. Cureus.

[24] Guo X., Guo S., Miao Z., Li Z., Zhang H. (2018). Myo-Inositol Lowers the Risk of Developing Gestational Diabetic Mellitus in Pregnancies: A Systematic Review and Meta-Analysis of Randomized Controlled Trials with Trial Sequential Analysis. J. Diabetes Complicat..

[25] Zyla K., Mika M., Dulinski R., Swiatkiewicz S., Koreleski J., Pustkowiak H., Piironen J. (2012). Effects of Inositol, Inositol-Generating Phytase B Applied Alone, and in Combination with 6-Phytase A to Phosphorus-Deficient Diets on Laying Performance, Eggshell Quality, Yolk Cholesterol, and Fatty Acid Deposition in Laying Hens. Poult. Sci..

[26] Qian J.Y., Kuhn M. (1999). Evaluation on Gelatinization of Buckwheat Starch: A Comparative Study of Brabender Viscoamylography, Rapid Visco-Analysis, and Differential Scanning Calorimetry. Eur. Food Res. Technol..

[27] Duliński R., Cielecka E.K., Pierzchalska M., Żyła K. (2015). Phytases Improve Myo-Inositol Bioaccessibility in Rye Bread: A Study Using an in Vitro Method of Digestion and a Caco-2 Cell Culture Model. Food Technol. Biotechnol..

[28] Duliński R., Starzyńska-Janiszewska A., Stodolak B., Zyla K. (2011). Comparison of High-Performance Ion Chromatography Technique with Microbiological Assay of myo-Inositol in Plant Components of Poultry Feeds. J. Anim. Feed Sci..

[29] Dionex—Thermo Fisher Scientific Inc. (2003). Application Note 65: Analysis of Inositol Phosphates.

[30] Blaabjerg K., Hansen-Møller J., Poulsen H.D. (2010). High-Performance Ion Chromatography Method for Separation and Quantification of Inositol Phosphates in Diets and Digesta. J. Chromatogr. B.

[31] Zyła K., Grabacka M., Pierzchalska M., Duliński R., Starzyńska-Janiszewska A. (2013). Effect of Inositol and Phytases on Hematological Indices and α-1 Acid Glycoprotein Levels in Laying Hens Fed Phosphorus-Deficient Corn-Soybean Meal-Based Diets. Poult. Sci..

[32] Qiu R., Lu J. (2017). Improved Hydrolase Activity in Barley and Reduced Malting Time by Adding Phytase as an Activator during Malting Steeping. Biotechnol. Lett..

[33] Kordialik-Bogacka E., Bogdan P., Ciosek A. (2019). Effects of Quinoa and Amaranth on Zinc, Magnesium and Calcium Content in Beer Wort. Int. J. Food Sci. Technol..

[34] Raes K., Knockaert D., Struijs K., Van Camp J. (2014). Role of Processing on Bioaccessibility of Minerals: Influence of Localization of Minerals and Anti-Nutritional Factors in the Plant. Trends Food Sci. Technol..

[35] De Nicola R., Walker G.M. (2011). Zinc Interactions with Brewing Yeast: Impact on Fermentation Performance. J. Am. Soc. Brew. Chem..

[36] Walker G., Stewart G. (2016). Saccharomyces Cerevisiae in the Production of Fermented Beverages. Beverages.

[37] Muy-Rangel D., Rubio-Carrasco W., Contreras-Angulo L. (2018). Differences in Physicochemical, Mineral and Nutraceutical Properties between Regular, Light and Zero Beers. Farmacia.

[38] Lazarte C.E., Carlsson N.G., Almgren A., Sandberg A.S., Granfeldt Y. (2015). Phytate, Zinc, Iron and Calcium Content of Common Bolivian Food, and Implications for Mineral Bioavailability. J. Food Compos. Anal..

